# Targeting the Inositol Pyrophosphate Biosynthetic Enzymes in Metabolic Diseases

**DOI:** 10.3390/molecules25061403

**Published:** 2020-03-19

**Authors:** Sandip Mukherjee, Jake Haubner, Anutosh Chakraborty

**Affiliations:** Department of Pharmacology and Physiology, Saint Louis University School of Medicine, Saint Louis, MO 63104, USA; sandip.mukherjee@health.slu.edu (S.M.);

**Keywords:** IP6, 5-IP7, IP6K, obesity, diabetes, NAFL

## Abstract

In mammals, a family of three inositol hexakisphosphate kinases (IP6Ks) synthesizes the inositol pyrophosphate 5-IP7 from IP6. Genetic deletion of *Ip6k1* protects mice from high fat diet induced obesity, insulin resistance and fatty liver. IP6K1 generated 5-IP7 promotes insulin secretion from pancreatic β-cells, whereas it reduces insulin signaling in metabolic tissues by inhibiting the protein kinase Akt. Thus, IP6K1 promotes high fat diet induced hyperinsulinemia and insulin resistance in mice while its deletion has the opposite effects. IP6K1 also promotes fat accumulation in the adipose tissue by inhibiting the protein kinase AMPK mediated energy expenditure. Genetic deletion of *Ip6k3* protects mice from age induced fat accumulation and insulin resistance. Accordingly, the pan IP6K inhibitor TNP [N2-(*m*-trifluorobenzyl), N6-(*p*-nitrobenzyl)purine] ameliorates obesity, insulin resistance and fatty liver in diet induced obese mice by improving Akt and AMPK mediated insulin sensitivity and energy expenditure. TNP also protects mice from bone loss, myocardial infarction and ischemia reperfusion injury. Thus, the IP6K pathway is a potential target in obesity and other metabolic diseases. Here, we summarize the studies that established IP6Ks as a potential target in metabolic diseases. Further studies will reveal whether inhibition of this pathway has similar pleiotropic benefits on metabolic health of humans.

## 1. Introduction

**Figure 1:**
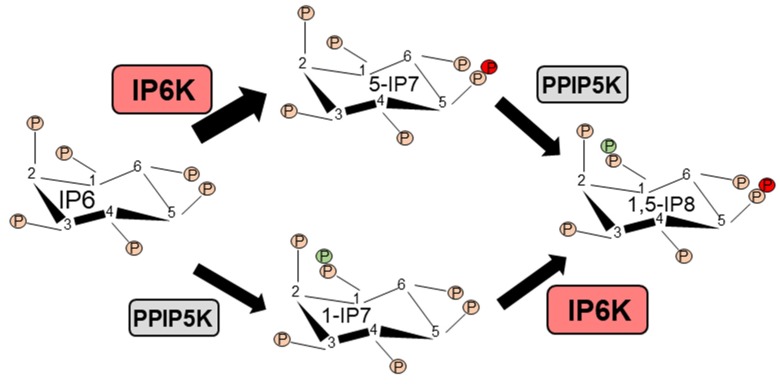
IP6Ks generate 5-IP7 from IP6. PPIP5Ks synthesize 1,5-IP8 from 5-IP7. The right panel shows the alternate route of the synthesis of 1,5-IP8. Left panel. 5-IP7 is the most abundant and characterized inositol pyrophosphate in mammals.

Inositol phosphate (IP) derivatives with energetic di-(β)-phosphates of IP6 (inositol hexakisphosphate or phytic acid) were identified in the early 1990s [1,2,3,4]. These biomolecules were named ‘inositol pyrophosphates’ (IPPs) to differentiate them from the monoester-based IPs [1,2,3,4,5,6,7]. The complex nomenclature of IPPs has been discussed previously [8,9,10,11,12,13]. Four major types of IPPs are characterized in mammalian cells; 5-diphosphoinositol (1,3,4,6)-tetrakisphosphate (5PP-IP4), 1-diphosphoinositol (2,3,4,5,6) pentakisphosphate (1PP-IP5 or 1-IP7), 5-diphosphoinositol (1,2,3,4,6) pentakisphosphate (5PP-IP5 or 5-IP7) and 1,5-bisdiphosphoinositol (2,3,4,6) tetrakisphosphate (1,5PP2-IP4 or 1,5-IP8), of which 5-IP7 is most characterized ([12] and references therein). A family of three inositol hexakisphosphate kinases (IP6K1-3) produces 5-IP7 by incorporating a phosphate group specifically to ‘position 5′ of the inositol 1,2,3,4,5,6-hexakisphosphate (IP6). Similarly, IP6Ks generate 5PP-IP4 from the inositol 1,3,4,5,6-pentakisphosphate (IP5) [14]. IP6Ks exhibit a high *Km* value toward ATP, and thus a higher cellular ATP/ADP ratio is required to synthesize 5-IP7 [10]. Conversely, at a lower ATP/ADP ratio, IP6Ks dephosphorylate IP6 to a distinct form of IP5 [I(2,3,4,5,6)P5 or IP5*] [15]. Another family of enzymes, the diphosphoinositol pentakisphosphate kinases (PPIP5K1 and PPIP5K2) similarly synthesizes 1-IP7 from IP6 [16,17,18]. IP6Ks or PPIP5Ks phosphorylate 1-IP7 or 5-IP7, respectively, to generate 1,5-IP8, which is undetectable under basal conditions. However, in the human colon cancer cell line HCT116, 10-20% of total IP7 can be converted to 1,5-IP8 [12,19]. Figure 1 presents the major inositol pyrophosphate synthetic pathway in mammals.

Disruption of IP6Ks reduces IPPs like 5PP-IP4, 5-IP7 and 1,5-IP8 and the inositol pentakisphosphate IP5*, depending on the cell’s energy status. The IPPs are hydrolyzed by the diphosphoinositol polyphosphate phosphohydrolase (DIPP) enzymes, which belong to the *Nudt* gene family [20]. Dependence of IP6Ks on the cellular ATP/ADP ratio partly explains the higher levels of IPPs in anabolic conditions [10,21]. Overnight serum starvation decreases the IP7 level in mouse embryonic fibroblast (MEF) or human hepatocellular carcinoma (HepG2) cells, which is restored by exposure to insulin-like growth factor-1 (IGF-1) or insulin [22]. Similarly, the IP7 level is increased during adipogenesis, which is also an anabolic process [22]. IPPs regulate cellular processes by modulating protein targets by binding or by adding its β-phosphate on the phosphate group of already phosphorylated proteins (pyrophosphorylation). IP6Ks may or may not interact with target proteins to facilitate 5-IP7-mediated effects. IP6Ks also regulate certain protein targets by direct protein-protein interaction, which is not dependent on their catalytic activity [12]. In addition to the classic lipid-IP3 pathway (phosphatidylinositol 4,5-bisphosphate, PIP2 is cleaved to diacylglycerol and inositol 1,4,5-trisphosphate IP3. IP3 gets converted to higher IPs and IPPs), a soluble route also exists where conversion of the glucose-6-phosphate to IP1 serves as a precursor of higher IPs and IPPs [23]. Additional details of IPPs, their metabolizing enzymes, functions and mechanism of actions have been reviewed ([8,12,14,18,20,23,24,25,26,27,28] and references therein).

A major focus of the IPP research is to determine in vivo significance of these molecules [12,25]. Development of various knockout mouse models and pharmacologic inhibitors of IP6Ks greatly enhanced this effort [29,30,31,32,33,34,35]. Expression analysis revealed that *Ip6k1* mRNA predominates in most mouse tissues with highest expression in brain and testis [34,35,36]. In humans, both *Ip6k1* and *Ip6k2* predominate with *Ip6k2* expression being slightly higher in mammary gland, thymus, colon, adipose tissue, testis, prostate and smooth muscle. *Ip6k3* is minimally expressed in murine tissues with exceptions in heart, skeletal muscle and brain [32,34]. *Ip6k3* is the major form in murine and human skeletal muscle [34] and is expressed at similar levels to *Ip6k1* and *Ip6k2* in the thyroid. *Ip6k3* is the primary form in the human but not murine heart. Thus, isoform-specific expression patterns of *Ip6ks* are observed, which seem to slightly vary between mice and humans. As *Ip6k1* is the major murine isotype, *Ip6k1-KO* mice were characterized first, followed by *Ip6k2* and *Ip6k3* knockouts ([12,25], and references therein), whereas PPIP5Ks are currently being studied [37,38,39]. Genetic deletion of *Ip6k1* or *Ip6k3* or pharmacologic disruption of IP6Ks protects mice from metabolic diseases including obesity, type-2 diabetes (T2D), non-alcoholic fatty liver (NAFL), osteoporosis, myocardial infarction, ischemia reperfusion injury and aging [22,29,34,40,41,42,43,44,45]. These discoveries drew the attention of pharmaceutical companies. Takeda Pharmaceuticals recently developed new class of potent IP6K inhibitors with strong anti-diabetic and anti-cardiomyopathic effects in rodents (patent - WO2018182051). Thus, pharmacologic targeting of IP6Ks is expected to have pleiotropic benefits on human metabolic health [12].

This review starts with a summary of the literature that established IP6Ks as a potential target in metabolic diseases. It discusses the mechanisms by which IP6Ks promote weight gain and insulin resistance and how IP6K1 activity regulates the cross-talk among metabolic tissues in healthy and metabolic disease conditions. It also analyzes effects of the IP6K substrate IP6 in metabolism and provides information about IP6Ks in human metabolic diseases. The review ends by discussing the potential risks of IP6K inhibition and future directions of IP6 and IPP research to improve metabolic health.

## 2. Disruption of IP6Ks Ameliorates Metabolic Diseases

### 2.1. Obesity, Insulin Resistance and NAFL

Obesity, T2D and NAFL display common metabolic aberrations. Obesity is a global epidemic, which has nearly tripled between 1975 and 2016. It is associated with ~2.8 million annual deaths worldwide. Moreover, childhood obesity has become one of the major health challenges of the 21st century. In addition to high-income countries, obesity is now also prevalent in low-income countries (https://www.who.int/news-room/fact-sheets/detail/obesity-and-overweight). In 2017–2018, 42.4% of the US population suffered from obesity (https://www.cdc.gov/obesity/data/adult.html). Obesity coupled with T2D promotes ‘metabolic syndrome’ that includes insulin resistance, elevated triglycerides, blood pressure and adiposity and reduced HDL-cholesterol [46,47]. This spectrum enhances risks of various comorbidities like osteoarthritis, kidney failure, blindness, limb amputation, cardiovascular diseases, neurodegeneration, sleep apnea, and cancer [48,49,50]. In the US, obesity-related cancers account for 40% of all cancers [51]. Obesity and T2D are also associated with increased risks of developing non-alcoholic fatty liver disease (NAFLD) and steatohepatitis (NASH) [52,53]. In the US, the number of NAFLD cases is projected to increase from 83.1 million (2015) to 100.9 million (2030) of which ~27% will have NASH. This will create a major economic burden due to cirrhosis and hepatocellular carcinoma (HCC) related liver transplantation [53,54,55,56,57,58]. Obesity associated liver diseases initiate with benign accumulation of fatty acids and cholesterol (NAFL) [53]. With the progression of obesity, NAFL combines with inflammation and fibrosis to develop NAFLD/NASH. A subset of patients develops cirrhosis and HCC [53,54,56,59]. In obese individuals, dysfunctional adipocytes release inflammatory cytokines that promote insulin resistance and reduce the fat-storing ability of these cells. This increases the concentration of free fatty acids in the circulation. The liver accumulates fat by taking up the free fatty acids [53,54] and by increased *de novo* lipogenesis from dietary sugars [53,54,60,61].

A combination of drugs and lifestyle intervention is ideal for obesity [62,63,64,65,66]. An anti-obesity drug should also prevent or delay other metabolic diseases, as even a 5–10% weight loss significantly reduces risks of cardiovascular and kidney diseases, osteoarthritis and NAFLD/NASH [67,68]. According to the Food and Drug Administration (FDA) guidelines, a compound that decreases body weight by 5% over a long period without causing significant side-effects, should be encouraged as a potential anti-obesity drug [67]. Unfortunately, current FDA approved anti-obesity medications like orlistat, lorcaserin, phentermine-topiramate, naltrexone-bupropion and liraglutide have many restrictions and side-effects [65,67,69,70]. For these reasons, an ideal anti-obesity drug has a projected market of $3.7 billion [71]. Moreover, no approved treatments exist for NASH. Therefore, research is ongoing to identify new targets to develop safer and more effective anti-obesity and anti-NASH drugs by improving energy metabolism, insulin sensitivity and inflammation [53,54,55,56,72,73,74,75,76,77,78,79].

*Ip6k1-KO* mice are protected from obesity, insulin resistance and NAFL. Young, chow-fed *Ip6k1-KO* mice display slightly reduced body weight compared to *WT* [30]. This phenotype is robust in middle-aged (10-month old) knockouts, as they weigh substantially less due to reduced accumulation of fat [22]. Moreover, high fat diet-fed *Ip6k1-KO* mice gain about one-third of *WT*’s body and fat weight [22]. Thus, deletion of *Ip6k1* protects mice from obesity [12,80]. IP6K1-generated 5-IP7 promotes insulin secretion from the pancreatic β cells [81]. Accordingly, young, chow-fed *Ip6k1-KO* mice display lower plasma insulin but similar blood glucose level compared to *WT*, indicating mild insulin hypersensitivity in the knockouts [30], which is evident in middle-aged *Ip6k1-KO* mice [22]. The knockouts are also protected from high fat diet induced hyperinsulinemia, hyperglycemia, hypertriglyceridemia and NAFL [22,40]. High-fat diet induced hepatotoxicity is evidenced by increased serum levels of aspartate aminotransferase (AST) and lactate dehydrogenase (LDH) [53,82], which are reduced in *Ip6k1-KO* mice [22]. *Ip6k1-KO* mice also display improvements in various other metabolic parameters (Table 1).

Increased energy expenditure protects *Ip6k1-KO* mice from obesity, insulin resistance and NAFL. Body weight is maintained by the homeostasis of intake, absorption and expenditure of food energy. Energy is utilized during physiological and metabolic functions and physical activities [83]. A small portion is lost in excretion. The remaining energy is lost as heat during digestion, absorption, exercise, and environmental changes like cold temperature [83]. An alteration in the energy homeostasis leads to weight gain or loss [83,84,85]. Cold and diet-induced heat production that occurs primarily in the skeletal muscle and brown adipose tissue (BAT), is known as adaptive thermogenesis. This process is distinct from other tissues, and therefore is being targeted in obesity [83]. White adipocytes can also transform into brown-like cells [78,86,87,88]. An increase in BAT and/or an enhancement in white adipose tissue (WAT) browning augments energy expenditure and fat loss in rodents and humans [78,79,83,88,89,90]. In mice, the inguinal WAT (IWAT) depot undergoes browning most efficiently [78]. Various stimuli including cold-induced norepinephrine, thyroid hormone, and cardiac natriuretic peptides stimulate browning and thermogenesis via the cyclic AMP-GMP/protein kinase A (cAMP-cGMP/PKA) mediated induction of the mitochondrial uncoupling protein 1 (UCP1) [88,91]. Adipose tissue browning can be induced or diminished by reducing (to 4–6 °C) or increasing (to ~30 °C) the ambient (23 °C) temperature [88,92].

Energy intake is unaltered in *Ip6k1-KO* mice [22,30,40], which indicates that the knockouts are lean due to increased energy expenditure. Cold exposure reduces *Ip6k1* expression in the IWAT, indicating that the enzyme is not required during thermogenesis or inhibits the process [29]. Cold-induced energy expenditure is higher in *Ip6k1-KO* mice, which supports this possibility [40]. Accordingly, the IWATs, which are white-colored in *WT*, appear brownish in *Ip6k1-KO* mice and the knockout-IWAT express more UCP1 and other markers compared to *WT* [40]. Although the knockouts are protected from weight gain under all the temperature conditions tested, they gain more body weight at thermoneutral than at ambient temperature. Thus, *Ip6k1-KO* mice are protected from high fat diet induced obesity partly due to increased adipocyte browning mediated thermogenesis [22,40]. Thermoneutrality delays but does not completely abolish leanness in *Ip6k1-KO* mice, indicating IP6K1 also regulates energy expenditure in other organs [40]. High fat diet-fed adipocyte-specific *Ip6k1-KO* (*AdKO*) mice are also lean, but to a lesser extent than *Ip6k1-KO* mice. Moreover, thermoneutrality largely abolishes leanness in *AdKO* mice [29]. *AdKO* mice are also protected from hyperinsulinemia, hyperglycemia and NAFL, indicating that improved adipocyte metabolism indirectly protects them from these aberrations [29]. In summary, whole body *Ip6k1*-deletion protects mice from obesity, T2D and NAFL due to increased energy expenditure. IP6K1 inhibits adipocyte browning mediated energy expenditure, which partially impacts whole body energy metabolism.

The targets of IP6K1 in obesity, T2D and NAFL. Disruption of IP6Ks reduces intracellular 5-IP7 and increases the IP6 level [12,22,93]. Thus, the observed phenotypes in IP6K-disrupted mice may arise from a decrease in 5-IP7 or an increase in IP6 or both. In cells, IP6 or 5-IP7 modulates various protein targets to regulate glycolysis, vesicular trafficking, chromatin remodeling, DNA damage, cell migration and neutrophil function, which may directly or indirectly alter energy metabolism in vivo [12,25,28,94,95,96]. Moreover, IP6K1 regulates various targets by protein-protein interactions that do not require its catalytic activity [12,97,98]. These targets/pathways will not be discussed here for the following reasons; (i) impacts of the above processes in IP6K-disrupted mouse models have not yet been determined; (ii) protein-protein interactions may not be affected by pharmacologic agents that inhibit IP6K’s catalytic activity, and hence are not therapeutically significant. The following section describes two major pathways that catalytically active IP6K1 regulates to modulate energy metabolism in vivo.

IP6K1 inhibits the energy expenditure stimulating protein kinase AMPK. The decreased cellular energy status increases the AMP/ATP ratio, which activates the AMP-activated protein kinase (AMPK). AMPK stimulates the catabolic pathways to generate ATP while conserving the remaining ATP by switching off the anabolic pathways [99]. AMPK stimulates energy expenditure [29,100,101,102,103,104,105,106,107,108,109], which maintains normal body weight, insulin sensitivity and liver-metabolism [103,110,111,112,113,114,115,116,117]. Deregulation of AMPK is observed in obesity, diabetes and other metabolic diseases [29,100,101,102,103,104,105,106,107,108,109,110,111,112,113,114,115,117]. Accordingly, direct or indirect pharmacologic AMPK activators reduce obesity, insulin resistance and NAFLD and NASH [110,112,118,119,120,121,122,123,124]. Metformin, the most prescribed anti-diabetic drug that suppresses hepatic glucose production [125,126], exerts its effects partly via indirect activation of AMPK [110]. AMPK activation by metformin, AMP-analog AICAR or A769662 diminishes blood glucose levels [110,127,128]. Moreover, AICAR or the plant-derived alkaloid berberine enhance AMPK-mediated energy expenditure [101,108,129]. AMPK also reduces NAFLD and NASH via mechanisms that involve adipose tissue, liver, muscle and gut [110,112,118]. Metformin improved NAFLD in some clinical trials [119,120,121]. Moreover, the direct AMPK activator [122] CNX-012-570 enhances adipose tissue browning mediated energy expenditure, reduces body weight and NAFLD in mice [123]. PF-06409577 lowers lipid/cholesterol, and reduces hepatic lipid-biosynthetic/fibrotic genes in rodents and primates [124]. PXL770 (Poxel Pharma, Lyon, France) is in Phase-2a trials for NASH. Thus, targeting AMPK activation has therapeutic importance in obesity [103,123], T2D [130], NASH [118,131] and cardiovascular diseases [110,132,133,134].

AMPK stimulates fatty acid oxidation, glucose uptake, glycolysis and mitochondrial biogenesis by regulating acetyl CoA carboxylase (ACC), AKT substrate 160/glucose transporter (AS160/GLUT), phosphofructokinase B (PFKB) and peroxisome proliferator-activated receptor gamma co-activator 1α (PGC1α), respectively. Conversely, AMPK inhibits fatty acid biosynthesis, lipogenesis, cholesterol synthesis and gluconeogenesis by modulating ACC, sterol regulatory element binding protein 1C (SREBP1C), 3-hydroxy-3-methylglutaryl-CoA reductase (HMGCR) and CREB-regulated transcription co-activator 2 (CRTC2) or forkhead box transcription factors (FoxO1), respectively [99]. Moreover, AMPK promotes adipose tissue browning mediated thermogenic energy expenditure [116]. For example, various agents like apelin, miRNA-455, resveratrol, cryptotanshinone, medicarpin and AICAR increase AMPK activity and adipocyte browning [116]. The AMPK activator A-769662 reduces weight gain by increasing browning mediated energy expenditure in high fat diet fed mice [135]. Conversely, removal of AMPK in mouse adipocytes reduces cold tolerance and non-shivering thermogenesis in the BAT, and subsequent development of NAFLD and insulin resistance [112]. The effects of a β3-adrenergic agonist on the induction of BAT thermogenesis and the browning of white adipose tissue (WAT) are also blunted in mice lacking adipocyte-AMPK [112].

IP6K1 and AMPK activities are differentially regulated by the cellular energy status, as the increased AMP/ATP ratio activates AMPK but reduces 5-IP7 in cells. Conceivably, IP6K1 antagonizes AMPK’s actions. Indeed, *Ip6k1* deletion augments AMPK-mediated energy expenditure [29]. Cold-induced activation of AMPK is higher in the IWAT of adipocyte-specific *Ip6k1-KO* (*AdKO*) mice. Increased beige adipogenesis of *AdKO*-preadipocytes is diminished by *AMPK* depletion. Moreover, the pan IP6K inhibitor TNP [N2-(*m*-trifluorobenzyl), N6-(*p*-nitrobenzyl)purine] [35] enhances AMPK phosphorylation and activity in beige adipocytes and in the IWAT of high fat diet-fed mice [29]. AMPK consists of the catalytic α-and the regulatory β and γ subunits [136]. Liver kinase B1 (LKB1) or calcium calmodulin dependent protein kinase β (CAMKKβ) phosphorylates AMPKα at the threonine-172 (T172) residue, leading to its activation [133,137,138]. IP6, but not 5-IP7 stimulates LKB1-mediated phosphorylation of AMPKα. Catalytically active IP6K1 abrogates IP6′s stimulatory effect on AMPK phosphorylation, suggesting that conversion of IP6 to 5-IP7 reduces AMPK activity. Accordingly, TNP, which reduces intracellular 5-IP7 [15,22,35,43,44,139] and increases IP6 levels [22,93], enhances AMPK activity and energy expenditure in adipocytes [40]. Enzymes that generate and/or metabolize IP6 may also regulate AMPK. For example, IPMK generates IP4 and IP5, which are precursors of IP6, and thus, *Ipmk* deletion reduces IP5 and IP6 levels [140,141]. Metformin-induced AMPK stimulatory phosphorylation and activity are impaired in *Ipmk*-deleted MEF cells, which are restored by overexpression of catalytically active IPMK [140]. Therefore, the ratio of IP6/5-IP7 in a cellular microenvironment seems to regulate AMPK activity and energy expenditure.

IP6K1 inhibits the insulin sensitizing protein kinase Akt. The insulin receptor maintains energy homeostasis by stimulating various downstream pathways including the phosphoinositide 3-kinase (PI3K)/protein kinase B (PKB or Akt) pathway [117], and thus its aberration is observed in metabolic diseases [117,142,143,144,145,146,147,148,149,150]. Akt is a serine/threonine protein kinase that regulates numerous cellular processes including metabolism [117,144]. There are three isotypes of Akt (Akt1, 2 and 3) of which Akt1 and Akt 2 predominate in metabolic tissues [151]. The role of Akt isotypes in metabolic diseases has been reviewed elsewhere [117,151]. Akt enhances glucose uptake, increases glycogen synthesis and inhibits gluconeogenesis primarily by phosphorylating the AS160, glycogen synthase kinase 3β (GSK3β) and forkhead box protein O1 (FoxO1), respectively. AKT also promotes *de novo* lipogenesis and cholesterol production by regulating lipogenic genes, such as SREBP1c and peroxisome proliferator-activated receptor gamma (PPARγ) [117,142]. Akt is required for the development of BAT [152]. Moreover, the PI3K/Akt pathway promotes brown adipogenesis [153], UCP1 expression and glucose uptake [148], which augment energy expenditure, inhibit weight gain and improve insulin sensitivity in diet-induced obese mice [154]. Furthermore, the insulin sensitizing adipokine adiponectin (AdipoQ) enhances browning via promoting PI3K/Akt mediated proliferation of M2 macrophages [155]. So, Akt reduces blood glucose level, whereas it may increase or decrease fat mass, depending on the physiological context.

In unstimulated conditions, Akt is inactive due to the intramolecular interaction between its pleckstrin homology (PH) and kinase domains. Insulin or other growth factor stimulated receptor tyrosine kinases (RTKs) activate the class-I PI3K, which phosphorylates phosphatidylinositol 4,5-bisphosphate (PIP2) to phosphatidylinositol 3,4,5-trisphosphate (PIP3) at the plasma membrane. This triggers Akt’s membrane-translocation, where its PH domain binds PIP3, which alters its conformation [156]. Subsequently phosphoinositide dependent kinase 1 (PDK1) and mammalian target of rapamycin complex 2 (mTORC2) phosphorylate Akt at threonine-308 (T308) [157] and serine-473 (S473) residues [144], respectively, leading to its full activation [158]. 5-IP7 inhibits Akt by interfering with Akt-PH domain’s binding to PIP3, which blocks its membrane translocation and PDK1 mediated phosphorylation [22,159,160,161]. IP6 also inhibits Akt [160,162,163], although to a lesser extent than 5-IP7 [22]. Consequently, Akt activity is higher in metabolic tissues of *Ip6k1*-deleted or IP6K-inhibited mice, especially in aged or high fat-fed conditions [22,29,42,43,44,45,139]. In hepatocytes, IP7 level increases with age, which reduces Akt activity [22]. High fat diet-fed *AdKO* mice display higher Akt activity not only in the EWAT, but also in liver and gastrocnemius muscle, which indicates that improved systemic insulin sensitivity and/or altered adipokine expression in adipocyte-specific *Ip6k1* deleted mice indirectly increase Akt activity in other tissues. Indeed, the plasma level of the insulin-sensitizing adipokine adiponectin (ADIPOQ) is higher in high fat diet-fed *AdKO* mice [29]. IP6K1/5-IP7 mediated regulation of Akt is complex in the pancreatic β cells. IP6K1 promotes insulin secretion, which activates Akt in β cells. Insulin’s profound stimulatory effect on Akt masks 5-IP7 inhibitory effect in these cells [164]. The status of Akt activity in the β cells of *Ip6k1-KO* mice, especially in aged or high fat-fed conditions is not known. *Ip6k3-KO* skeletal muscle and heart do not display increased Akt activity [34]. A single dose of TNP treatment increases insulin sensitivity and Akt activity in adipose tissue, liver and muscle of diet induced obese mice [42]. In summary, IP6K1/5-IP7 exerts direct and indirect inhibitory effects on Akt in peripheral metabolic tissues, which regulates insulin sensitivity.

The PI3K/Akt pathway is also frequently inhibited in obesity, diabetes and other metabolic diseases [117,142,143,144,145,146,147,148,149,150]. However, pharmacologic activation of this pathway has not been exploited to treat these diseases. The compound SC 79 activates Akt by binding to its PH domain, which enhances its phosphorylation at T308 by PDK1 [165]. SC 79-mediated Akt activation rescues ischemia-induced neuronal death in rodents [165,166]. It also protects rodents from bacterial lipopolysaccharide-induced liver injury [167,168] and hepatic [169], renal [170] and cerebral ischemia [166]. Determining effects of SC 79 in metabolic diseases, either alone or in combination with IP6K inhibitor of AMPK activator will be of interest. AMPK and Akt pathways display antagonism, especially in cancer cells ([171,172,173,174,175,176], and references therein). Therefore, the observed Akt and AMPK activation in *Ip6K1*-deleted and TNP-treated metabolic tissues has specific consequences in systemic metabolism, which may or may not be observed in other diseases like cancer.

*Ip6k3-KO* mice are protected from age induced weight gain and insulin resistance. In the murine skeletal muscle, *Ip6k3*’s mRNA expression positively correlates with impaired glucose metabolism like diabetes and disuse (unused muscle) conditions and fasting [34]. Energy intake is unaltered in chow-fed *Ip6k3-KO* mice, but they exhibit a lower body and fat mass from 51 weeks to 1.5 years of age. *Ip6k3* is undetectable in β cells or islets [34,81], yet, aged *Ip6k3-KO* mice display reduced plasma insulin levels, presumably due to increased insulin sensitivity. These phenotypes resemble middle-aged (10-months old) *Ip6k1-KO* mice [22].

The fast-glycolytic muscle fibers generate lactate, which is utilized by the liver during gluconeogenesis (Cori cycle). Thus, increased plasma lactate in *Ip6k3-KO* mice indicates enhanced muscle-glycolysis. *Ip6k3-KO*s show a decrease in ad libitum blood glucose level, possibly due to increased glucose uptake in the skeletal muscle (and in other tissues) to support glycolysis. Moreover, fasting blood glucose (0-time point, insulin tolerance test) [34], which is maintained by gluconeogenesis, is reduced in *Ip6k3-KO* mice. Presumably, the *Ip6k3-KO*-liver generates pyruvate from lactate, which is then directed to acetyl-CoA, but not to glucose. Reduced expression of the pyruvate dehydrogenase kinase-4 (PDK4), which inhibits pyruvate to acetyl-CoA conversion, in *Ip6k3-KO* muscle (liver data is not available) supports this possibility. Acetyl-CoA fuels mitochondrial oxidation or lipogenesis, depending on the cellular energy status. Seemingly, *Ip6k3-KO* mice oxidize acetyl-CoA, as they accumulate less lipid [34]. However, *WT* and *Ip6k3-KO* mice oxidize fatty acid generated acetyl CoA to a similar extent, as they gain similar body weight on high fat diet [34]. Further studies in myocyte and hepatocyte-specific *Ip6k3-KO* mice should be conducted to distinguish the role of IP6K3 in glucose uptake, glycolysis, glucose oxidation, gluconeogenesis and lipogenesis. Nevertheless, these studies demonstrate that IP6K3 can be targeted in age induced metabolic dysfunction.

Pharmacologic inhibition of IP6Ks ameliorates obesity, insulin resistance and NAFL. Studies in preclinical rodent models demonstrated that the IP6K pathway is a potential pharmacologic target in obesity. The pan IP6K inhibitor TNP [15,22,35,43,45,139] enhances energy expenditure and inhibits fatty acid biosynthesis in 3T3L1 adipocytes [40]. TNP is 70-fold more potent on IP6Ks over its other target IP3-3K [35]. Moreover, TNP does not influence the 1-IP7 generating enzyme PPIP5K [177] or 71 unrelated kinases [35]. Short-term (18 days) TNP treatment causes a marginal reduction while long-term (10-weeks) treatment causes a substantial loss in body and fat mass in high fat diet induced obese mice. Overall, TNP treatment causes ~20% decrease in body weight compared to vehicle treated mice. TNP does not alter food intake, but increases energy expenditure. TNP’s anti-obesity effects are impaired but not abolished at thermoneutral temperature conditions, which further indicates that IP6K1 regulates energy expenditure in organs other than adipose tissue. Although high fat diet-fed *Ip6k1-KO* mice gain less body weight than *WT* mice, they do gain some weight, which is not further reduced by TNP treatment. Hence, TNP reduces weight gain specifically via inhibition of IP6K1. Both short- and long-term treatments of TNP reduce hyperglycemia, hyperinsulinemia and insulin resistance in high fat diet induced obese *WT* mice [42]. In these conditions, TNP also ameliorates NAFL and serum levels of AST and ALT [42]. Recently, Takeda Pharmaceuticals, MA, USA) developed new classes of IP6K inhibitors (patent—WO2018182051), which reduce hyperglycemia in diabetic Zucker-fatty rats and show higher potency and efficacy than the anti-diabetic drug metformin (3–10 vs. 150 mg/kg BW, respectively) in reducing blood glucose levels. In summary, IP6Ks can be pharmacologically targeted in obesity, T2D and NAFL.

### 2.2. Osteoporosis

The incidence of osteoporosis is expected to increase in the coming decades due to aging and lifestyle changes. In 2014, 54 million US adults were affected by osteoporosis. Bone marrow derived mesenchymal stem/stromal cells (BMMSCs) are the predominant source of osteocytes and adipocytes in the bone marrow [41,178]. Age or obesity induced oxidative and other changes in the bone marrow favors adipogenesis over osteogenesis, leading to skeletal involution and marrow adiposity [179,180,181]. Thus, pathways that induce osteogenesis and/or reduce adipogenesis of BMMSC are potential therapeutic targets to treat obesity or age related bone diseases [41]. *Ip6k1,* the major *Ip6k*-isotype in BMMSC regulates stem cell fitness. Young *Ip6k1-KO* BMMSCs exhibit enhanced growth, survival, osteogenesis and hematopoiesis. Conversely, they show reduced adipogenesis, which further explains diminished adipose mass in these animals. *Ip6k1-KO* BMMSCs display reduced ROS level [41]. Accordingly, long-term TNP treatment increases yield and colony-formation capacity of MSCs in high fat diet induced obese mice [41,42]. TNP conserved trabecular bone, decreased marrow adiposity and protects mice from high fat diet induced BMMSC dysfunction. Age induced decline in the yields of BMMSC is also partly prevented by *Ip6k1* deletion. Although IP6K-disruption mediated metabolic improvements can secondarily improve stem cell fitness, alterations in the known regulators of this process are also observed. The growth arrest and apoptosis inducing protein p53 is reduced and the E3 ubiquitin ligase mouse double minute-2 homolog (MDM2) that degrades p53, is increased in *Ip6k1-KO* BMMSCs [41]. Insulin sensitizers like PPARγ antagonists increase obesity and fracture risk. This study suggests that IP6K inhibitors may have greater acceptability as anti-obesity and anti-diabetic drugs due to their beneficial effects on bone [12]. Table 1 summarizes the metabolic phenotypes of *Ip6k1-KO* [22,30,40,41], *AdKO* [29], *Ip6k3-KO* [34] and TNP-treated *WT* [42] mice.

### 2.3. Myocardial Infarction

Aged BMMSCs exhibit higher IP7 levels and increased apoptosis compared to young cells. TNP treatment reduces IP7 and apoptosis in aged BMMSCs. Levels of angiogenic factors such as vascular endothelial growth factor (VEGF), basic fibroblast growth factor (bFGF), IGF-1 and hepatocyte growth factor (HGF), which are decreased in aged BM-MSCs, are restored by TNP treatment [44]. Moreover, TNP-treated BMMSCs, when transplanted into infarcted hearts, display enhanced survival, which promotes their anti-apoptotic and pro-angiogenic efficacy in vivo. Thus, IP6Ks and 5-IP7 promotes age related vulnerability to hypoxic injury and paracrine deficiency of BMMSCs [45]. Decreased viability and impaired function of aged BMMSCs are critical roadblocks for their therapeutic applications. Targeting the IP6K pathway may improve functions of BMMSCs.

### 2.4. Ischemia Reperfusion (I/R) Injury

The cytokine oncostatin M (OSM), which works via OSM receptor activation, alleviates cardiac ischemic/reperfusion (I/R) injury by inhibiting cardiomyocyte apoptosis in leptin receptor-deficient obese and diabetic *db/db* mice [43]. OSM enhances survival, mitochondrial biogenesis and reduces IP7 levels in these mice. Conversely, OSM receptor knockout increases IP7, impairs mitochondrial biogenesis, insulin sensitivity and augments cardiomyocyte apoptosis, which aggravates cardiac I/R injury. TNP treatment reduces IP7, decreases the infarct size and cardiomyocyte apoptosis in *db/db* mice [43].

### 2.5. Thromboembolism

Hemostasis is the process of platelet aggregation to prevent and stop bleeding. Slow hemostasis increases the clotting time, known as diathesis. Conversely, abnormally fast clotting leads to thromboembolism, which can cause fatal diseases like stroke, pulmonary embolism, thrombosis, and myocardial infarction. The platelet aggregation time is delayed in *Ip6k1-KO* mice, which lengthens the plasma clotting time. Consequently, *Ip6k1-KO* mice display a longer tail bleeding time, and are protected from pulmonary thromboembolism. Polyphosphates (PolyPs), enriched in platelets, mediate platelet aggregation. 5-IP7 maintains the PolyP levels [182], and thus PolyPs are reduced in *Ip6k1-KO* platelets. Therefore, PolyP mediated clotting is impaired in the knockouts. Addition of PolyP but not 5-IP7 rescues clotting in sera derived from *Ip6k1-KO* mice, indicating that 5-IP7 modulates the process indirectly by regulating PolyP levels [183].

### 2.6. Lifespan

*Ip6k3-KO* mice display a significantly extended lifespan compared to *WT* [34]. A decrease in the phosphorylation status of the mTOR downstream effector ribosomal S6 protein is observed in the heart but not in the skeletal muscle of *Ip6k3-KO* mice, indicating diminished protein synthesis in the knockout heart. Inhibiting mTOR mediated S6 phosphorylation and protein synthesis by rapamycin has been demonstrated to prolong lifespan in mice [184]. However, the phosphorylation status of the upstream kinases S6K1 and Akt are unchanged *Ip6k3-KO*, suggesting that IP6K3 regulates S6 phosphorylation and possibly protein synthesis independent of the classic Akt/mTOR signaling pathway [34]. Moreover, why S6 phosphorylation is not decreased in the skeletal muscle, where *Ip6k3* expression is the highest, is not understood. Although the impact of IP6K1 on the cardiac mTOR signaling is not known, S6 phosphorylation is moderately increased in the skeletal muscle of *Ip6k1-KO* mice [22]. Thus, further studies are needed to distinguish tissue-specific functions of IP6K1 and IP6K3 in S6 phosphorylation and aging to determine whether pan or isotype-specific inhibition of this pathway enhances lifespan.

### 2.7. IP6K1 Generated 5-IP7 Regulates the Cross-talk Among Metabolic Tissues

How does IP6K1 mediated enhancement in insulin secretion and inhibition in insulin signaling fit in the context of metabolic diseases? Under normal conditions, insulin stimulates Akt mediated phosphorylation of FoxO1 to reduce gluconeogenesis [185], and activates the transcription factor SREBP1c to enhance *de novo* lipogenesis [186]. Obesity, T2D and NAFLD patients exhibit the classic triad of hyperinsulinemia, hyperglycemia and hypertriglyceridemia [187]. Hyperglycemia induces hyperinsulinemia. In this condition, the Akt-FoxO1 axis becomes insulin resistant, whereas the SREBP1c axis maintains insulin sensitivity. Consequently, uncontrolled gluconeogenesis and lipogenesis occur, leading to hypertriglyceridemia. These aberrations concomitantly cause systemic insulin resistance and metabolic dysfunction by affecting adipose tissue and muscle [187].

**Figure 2:**
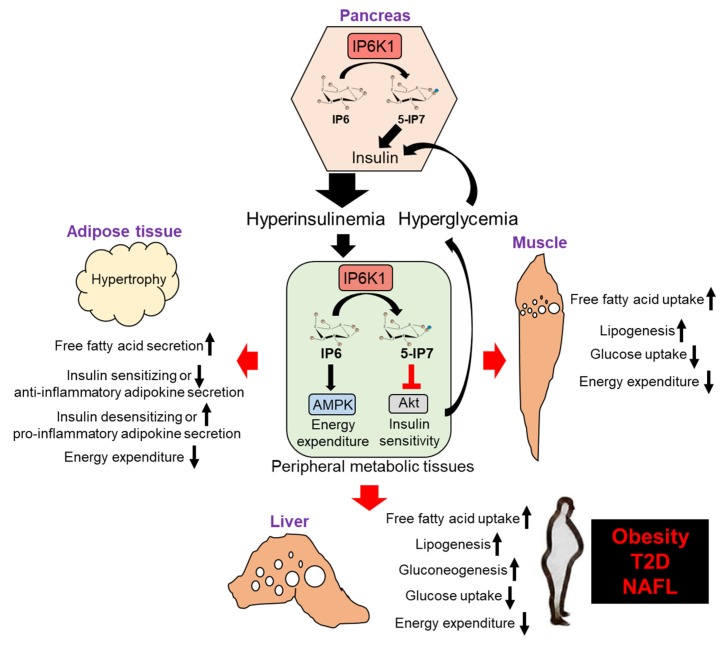
In obesity, 5-IP7 mediated inhibition of Akt causes insulin resistance in metabolic tissues (green box) causes hyperglycemia, which induces 5-IP7 mediated insulin secretion from pancreatic β cells (hexagon), resulting in hyperinsulinemia. Moreover, IP6K1 mediated conversion of IP6 to 5-IP7 reduces IP6′s stimulatory effects on AMPK mediated energy expenditure in the adipose tissue, leading to adipocyte dysfunction. Dysfunctional adipocytes secrete free fatty acids and insulin desensitizing and pro-inflammatory adipokines. Circulating free fatty acids are taken up by the liver from the serum. Although the rate of glucose uptake is lower in this condition, hyperinsulinemia causes a portion of glucose to enter the liver. Gluconeogenesis is also increased. Consequently, the liver accumulates fat (white droplets) by conversion of free fatty acids to triglycerides and *de novo* lipogenesis. The skeletal muscle also accumulates fat and becomes insulin resistant in a similar fashion. Therefore, increased IP6K1’s actions cause obesity, T2D and NAFL.

In healthy state, IP6K1’s anabolic functions promote energy storage, which is an important biological process. Scarcity of food during evolution created selective pressures favoring the conservation, and even duplication of genes that contribute to efficient energy storage. Triplication of the *Ip6k* gene from yeast to mammals suggests that this pathway may have evolved in this way [14,82]. Unfortunately, in the modern society, excess energy intake not only diminishes the need of its storage but also causes obesity. In obesity, IP6K1/5-IP7 mediated peripheral insulin resistance causes hyperglycemia, which further stimulates IP6K1/5-IP7 induced insulin secretion from β cells, resulting in hyperinsulinemia induced lipogenesis. Moreover, IP6K1 inhibits energy expenditure, which promotes hypertriglyceridemia [12,22,29,40,42]. Thus, IP6K1-disrupted mice are protected from these aberrations [22,42] (Figure 2). To summarize, in energy enriched conditions, targeting IP6Ks ameliorates obesity, T2D and other metabolic diseases.

### 2.8. Role of the IP6K Substrate IP6 in Metabolism

IP6, the substrate of IP6Ks is the most abundant IP in plants and mammalian cells [188]. IP6 research is divided into two major areas: studying the role of intracellular IP6 (Int-IP6) by manipulating the enzymes that synthesize or metabolize this molecule, and assessing the impact of dietary supplementation of IP6 (Sup-IP6) in health. Besides the already established PIP2 or IP3 [8,12,14,18,20,23,24,25,26,27], glucose-6-phosphate and sphingolipids can also be precursors of Int-IP6 [23]. Int-IP6 regulates many cellular functions including stress responses, development, phosphate homeostasis, DNA repair, RNA editing, mRNA export and post-translational modification [94,95,189]. It also promotes insulin secretion from β cells, although to a lesser extent than 5-IP7 [81,190]. Moreover, Int-IP6 regulates metabolism by activating AMPK [29].

The concentration of IP6 is much higher in plant sources, especially cereals, legumes, nuts and high-fiber diets, than in animal products [94,191,192,193]. Dietary IP6 may impact health in both positive and negative ways, the details of which are discussed elsewhere [94,192,193]. IP6’s negative impact is due to its cation (particularly iron and zinc)-chelating action, which hinders cation-absorption in the gastrointestinal tract [194,195]. Thus, in low-income countries where plant-based products with high IP6/iron or IP6/zinc ratios are the major food sources, mineral deficiencies often occur [192]. In developed countries, however, animal products also contribute to body’s iron and zinc contents. In such conditions, plant-derived dietary IP6 does not appear to cause any problem [94,193].

Studies in rodent models demonstrated that Sup-IP6 provides protection against various types of cancers, thrombosis, inflammatory bowel disease, Alzheimer’s disease, kidney stones and mycotoxins [94,191,196,197]. In a clinical study, breast cancer patients who received Sup-IP6+inositol displayed less side effects of the chemotherapy [198]. Sup-IP6 have also been shown to improve metabolism [199,200,201,202,203,204,205,206,207,208,209,210]. Here, the intricacies of sources, doses and durations of Sup-IP6 are not discussed, which can be found in the respective references. Western diets, which are associated with metabolic diseases, contain less IP6 than balanced healthy diets that contain more plant-based products [193]. The Sup-IP6 content correlates inversely with the glycemic index of healthy human subjects [200]. In a diabetic KK mouse model, Sup-IP6 reduces glucose and hemoglobin A1c levels and improves glucose tolerance without altering the insulin level, body weight or food intake [201]. Sup-IP6 reduces xenobiotic (DDT)-induced hypercholesterolemia [211] and oxidative stress induced liver injury in BALB/c mice [212]. In high fat diet and streptozotocin treated diabetic Sprague-Dawley rats, a combination of Sup-IP6 and Sup-inositol reduces insulin resistance and serum levels of glucose, triglycerides and total cholesterol. It also diminishes food intake via the satiety inducing adipokine leptin [213], whereas Sup-IP6 alone reduces weight gain, triglycerides and hyperglycemia, and increases food intake and total and HDL (good) cholesterol levels [210]. In streptozotocin-nicotinamide induced diabetic rats, Sup-IP6 decreases glucose, HbA1C, and lipid-peroxidation, whereas it enhances HDL and antioxidants in liver and small intestine. However, in this condition, Sup-IP6 increases body weight [207]. Sup-IP6 also ameliorates NAFLD by reducing liver weight, hepatic lipid and triglyceride levels, activity of lipogenic enzymes and serum levels of inflammatory cytokines in rats fed high sucrose or high fat diets [202,203,205,208]. In rats, it also protects against bone loss to lower the risk of osteoporosis [214,215] and reduces aorta calcification [216,217] and protects ischemic hearts from reperfusion injury [218].

Although it is not entirely clear how Sup-IP6 improves metabolism, it may work, at least in part, by inhibiting the enzymes alpha-amylase and glucosidase that breakdown starch to glucose [207]. Targeting these enzymes is a potential approach to treat obesity and diabetes, and thus their inhibitors are in clinical trials [219,220]. IP6 inhibits α-amylase and α-glucosidase in vitro [207]. Accordingly, IP6 reduces the rate of digestion of starch in saliva [200], whereas removal of Sup-IP6 increased the digestion of starch and raised the glycemic response in humans [209]. Sup-IP6 may increase Int-IP6 or the lower forms of IPs like IP5, IP3 or inositol, generated from IP6 [221]. Sup-IP6 increases IP6 levels in plasma and various tissues such as brain and liver [222,223]. Conversely, rats fed an IP6-free diet, display substantial reductions in the tissue IP6 levels, which is restored upon addition of Sup-IP6. However, one study failed to detect IP6 in the plasma [224]. The methods to detect IP6 in these experiments were different. Moreover, it is not known whether the subjects consumed diets that are rich or poor in IP6 [223]. Nevertheless, it can be presumed that unlike cell culture studies, where Int-IP6 originates only from biosynthesis, its level in tissues may depend on both dietary intake and biosynthesis [222,225]. Extracellular IP6 is internalized by the cells into the lysosomes possibly via non-receptor-mediated endocytosis, and gets slowly degraded to lower forms of IPs and free inositol [221,226]. Thus, it is not entirely clear whether Sup-IP6 works by increasing Int-IP6 or by increasing the lower forms of IPs. Further studies are needed to determine the mechanisms by which Int-IP6 and Sup-IP6 works. It is also crucial to determine to what extent the above-mentioned pleiotropic beneficial effects of dietary IP6 are translatable in humans. To summarize, a high IP6/cation ratio causes mineral deficiency, whereas IP6-supplementation in westernized diets may have beneficial effects in metabolic diseases.

### 2.9. IP6Ks in Human Metabolic Diseases

IP6Ks are highly conserved in rodents and humans, although isotype-specific expression may vary slightly [34]. Limited information is available on gene expression and polymorphism profiles of *Ip6ks* in diseases. An epigenome-wide association study identified higher CpG methylation upstream of *Ip6k1* in the saliva of overweight adolescent Finnish girls compared to lean girls [227], suggesting diminished expression of the gene in obesity. It is conceivable that *Ip6k1* expression is reduced in a natural negative-feedback mechanism. Disruption of *Ip6k1* at intron 1 was observed in a single Japanese family with T2D [228]. However, this anomaly was not found in 405 unrelated patients, indicating that the disruption is either family-specific or a chance association. The expression of *Ip6k1* correlates inversely with insulin sensitivity [229], whereas both *Ip6k1* and *Ip6k2* expression directly correlate with hepatocellular carcinoma (HCC) [230]. *Ip6k3* is upregulated in NAFLD patients [231]. The mRNA expression does not always correlate with protein or activity level [232], and so studies are required to determine activity, localization and protein interactions of these enzymes in tissues derived from healthy and sick subjects.

### 2.10. Potential Risks of Targeting the IP6K Pathway in Metabolic Diseases

Targeting any protein or pathway has potential risks as every protein is naturally evolved to perform certain essential functions. Therefore, it is vital to determine the exact conditions when targeting a specific protein is beneficial. In modern society, excess intake of unhealthy diets causes obesity, T2D, NAFLD/NASH and other metabolic diseases. Hence, the anabolic functions of IP6K1 can be targeted in metabolic diseases in the mid-late stage of life.

IP6K1 and IP6K3 are essential for development of brain and testis, and thus *Ip6k1-KO* mice exhibit impaired social interaction and male sterility [30,98,233,234], whereas *Ip6k3-KOs* display reduced motor learning and coordination [32]. However, TNP (15-weeks, 10 mg/kg, daily) treatment does not cause neuronal defects or male-sterility [42]. Thus, germline deletion mediated developmental defects does not seem to be a concern in pharmacologic targeting of this pathway at a later stage of life. Also, drugs do not permeate well through the blood-brain- or blood-testis- barriers (BBB or BTB), which impairs drug delivery to these organs [235,236]. Targeting IP6Ks may have differential impacts on neutrophils. Although disruption of IP6K1 reduces pulmonary neutrophil accumulation induced lung damage in pneumonia, its deletion aggravates lung inflammation in nicotine-exposed mice [237]. Delayed platelet aggregation in *Ip6k1-KO* mice [183] is beneficial in thromboembolism, but may delay clotting in normal subjects.

IP6K1 inhibits Akt in metabolic cells and tissues [12,29,40,42,43,44,45,238]. In young, chow-fed tissues, IP6K1-disruption minimally alters Akt [22,29], whereas its perturbation reduces high fat diet or age induced Akt inhibition. Thus, Akt activity is higher in IP6K-disrupted tissues compared to *WT* in these conditions [29]. Amplification or constitutive hyperactivation (~10–50-fold) [239,240] of Akt causes tumorigenesis in vivo [241], whereas no tumors are formed when these conditions are not met [241]. Notably, a 2–3-fold increase in Akt activation improves metabolic functions [12,22] without forming tumors [242] in *Ip6k1-KO* mice. Moreover, *Ip6k1-KO* mice are protected from tumorigenesis and metastasis [242]. Thus, regulated activation of Akt in metabolic diseases is distinct from its constitutive hyperactivation in cancer, which should be exploited to treat metabolic diseases. *Ip6k2* deletion sensitizes mice to 4-Nitroquinoline 1-oxide (4-NQO) induced aerodigestive tract carcinoma [33], but protects them from cancer metastasis [243]. Encouragingly, even long-term TNP treatment does not cause tumorigenesis. While developing pan IP6K or isotype-specific inhibitors to treat metabolic diseases, these potential concerns must be ruled out.

### 2.11. Outstanding Questions on Targeting IP6Ks in Metabolic Diseases

#### 2.11.1. Can IP6Ks be Targeted in NAFLD/NASH, HCC and Atherosclerosis?

Human NASH is associated with obesity, metabolic syndrome and fibrosis [57]. The published studies on IP6K1 used a high fat diet without added cholesterol or fructose [22,29,40,42] that develops obesity and NAFL, but not a full spectrum of NAFLD/NASH that includes inflammation, ballooning, stellate cell activation and fibrosis [53,54,55,56,244]. To develop liver injury and NASH, high cholesterol and/or fructose-containing Western diets are preferred. Moreover, mouse models of atherosclerosis are also used as NASH models because NASH and atherosclerosis share substantial similarities in the manifestation of metabolic syndrome [59,245]. Thus, Western diet-fed *apolipoprotein E* (*ApoE*) or *low density lipoprotein receptor* (*Ldlr*) knockout mice, which develop metabolic syndrome and atherosclerosis, also develop NAFLD/NASH [246,247,248,249,250,251,252,253,254,255,256,257,258,259,260,261]. *Ip6k3* is upregulated in NAFLD patients [231], whereas *Ip6k1* and *Ip6k2* expression directly correlate with hepatocellular carcinoma (HCC) [230]. Does disruption of IP6K protect mice from NASH, HCC and atherosclerosis? Further studies are needed to answer these questions.

#### 2.11.2. Can the Ratio of IP6/IPP be Targeted in Metabolic Diseases?

A reduction in 5-IP7 and/or an increase in IP6 level improves metabolism *in vivo*. Consequently, the ratio of IP6/5-IP7 (or other IPPs) seems to regulate energy metabolism like NAD^+^/NADH and AMP/ATP. Therefore, increasing the IP6/IPP ratio could be an interesting approach to treat metabolic and possibly other diseases. For example, activation of IP5K in the settings of IP6K- and/or PPIP5K-disruption seems like a feasible approach. It is also important to understand what regulates expression, stability activity and localization of IP5K or IP6Ks to alter the IP6/5-IP7 ratio in a cellular microenvironment. Various posttranslational modifications and protein interactors regulate IP6Ks ([97,98,262,263,264,265] and Chakraborty lab unpublished observations). Significance of the known and novel interactions and modifications are being studied.

If IP6 is abundant in cells, why is its further augmentation needed? Presumably, the abundance of IP6 keeps the IP6/IPP ratio high/optimum, which is required for the metabolic health of cells or tissues. However, in metabolic aberrations such as insulin resistance [229], HCC [230], NAFLD [231] and aging [22,44], *Ip6k* expression or IP7 level increases, which is expected to reduce the IP6/5-IP7 ratio. In such conditions, restoring this ratio to its normal level may ameliorate these diseases. To determine the ratio of IP6/5-IP7, a reliable method to quantify their levels in biological samples is required, but unfortunately not available yet. Encouragingly, various groups are working to solve this issue [19,224].

#### 2.11.3. Can IP6K Inhibitors Be Used to Treat Metabolic Diseases?

An important requirement for drugs to treat metabolic diseases is their long-term efficacy and safety. It is too early to comment whether inhibition of IP6Ks will achieve this goal. TNP treatment in mice for 15-weeks did not cause any noticeable side-effect [42], which is an encouraging start. Although TNP is an established pharmacologic tool, it has limitations that hinders its further development or for its use in the clinical trials [42]. TNP inhibits all the IP6K isotypes. It also inhibits human cytochrome P450s [42], which may interfere with the metabolism of other drugs. In addition, TNP slightly elevates Ca^2+^ levels in human promyelocytic leukemia (HL60) cells [266] and cytosolic Zn^2+^ levels in cortical neurons [267], which may cause signaling alterations. At higher concentrations, TNP inhibits the IP3-3 kinase [268,269]. TNP has a modest half-life in microsomal assays, suggesting a mediocre metabolic stability. The development of IP6K inhibitors with greater metabolic stability is required to achieve oral efficacy in patients. To minimize undesirable effects, isoform-selective inhibitors are needed. BBB- and BTB-impermeable IP6K1 (or IP6K1+IP6K3) selective inhibitors are desirable for the treatment of metabolic diseases. It is also necessary to determine the therapeutic window for each disease. Several groups are working to screen compound libraries against IP6Ks and to exploit subtle differences in active sites of the isotypes to develop potent and isotype-specific IP6K inhibitors [42,270,271,272]. Hopefully, IP6K inhibitors, either alone or in combination, will emerge as new drugs to treat metabolic diseases.

## 3. Conclusions

Studies in preclinical rodent models establish that targeting the 5-IP7 biosynthetic enzymes IP6Ks is a potential therapeutic approach to treat obesity, T2D, NAFL, osteoporosis and other metabolic diseases. Methods to increase the ratio of IP6/5-IP7 may also emerge as a therapeutic strategy in metabolic diseases. Conversely, IP6Ks are essential for the development or brain and testis, insulin secretion and energy storage. Thus, diseases associated with energy or insulin deficiency such as lipodystrophy, cachexia and type-1 diabetes or brain/testis development, overexpression of this pathway may have beneficial effects. Limited information is available on IP6Ks in humans. Therefore, research should be directed to understand the importance of this pathway in human health and diseases.

## Figures and Tables

**Table 1:** Summary of the metabolic parameters of *Ip6k1-KO* [22,30,41], *AdKO* [29], *Ip6k3-KO* [34] and TNP-treated *WT* [42] mouse models in ambient temperature conditions. Symbols ↑, ↓ and = denote increase, decrease and no change, respectively. # indicates that the study was done in 18-month old *Ip6k1-KO* mice instead of middle-aged (10-month) mice. Blank denotes that the parameter was not measured. Parameters that were measured in only one model are not included. For *Ip6k1*-KO [40] and *AdKO* [29] mice, some parameters were assessed in thermoneutral conditions, which are not included.

	*Ip6k1-KO*	*Ip6k1-KO*	*Ip6k1-KO*	*AdKO*	*Ip6k3-KO*	WT+TNP
	Adult-chow	Adult-HFD	Middle-Aged-Chow	Adult-HFD	Aged-Chow	Adult-HFD
**Body and Fat Mass**						
Body mass	↓	↓	↓	↓	↓	↓
Fat mass (Total)	↓	↓	↓	↓	↓	↓
Lean mass (total)	=	↓	↓	↓	=	=
Fat mass (% total body weight)	↓	↓	↓	↓		↓
Lean mass (% total body weight)	↑	↑	↑	↑		↑
Energy Expenditure	=	↑		↑		↑
Food Intake	=	=		=		=
**Serum Metabolic Parameters**						
Insulin	↓	↓	↓	↓	↓	↓
Glucose	=	↓	↓	↓	↓	↓
Insulin Sensitivity	↑	↑	↑	↑	↑	↑
Cholesterol (total)	=	↓	↓			↓
Triglycerides	↓	↓				=
AST	=	↓	=			↓
ALT			=			=
LDH	=	↓				
Adiponectin		↑		↑		
Leptin	↓	↓				
**Adipose Tissue/Adipocytes**						
Adipose tissue weight	↓	↓	↓	↓		↓
Adipocyte size	↓	↓	↓	↓		↓
Browning/UCP1 expression	↑	↑		↑		↑
Oxygen consumption rate	↑			↑		
**Liver**						
Liver weight	=	↓		↓		↓
Liver fat	↓	↓	↓	↓		↓
**Bone Marrow Stem Cells**						
Survival and yield	↑		↑#			↑
Adipogenesis/marrow adiposity	↓		↓#			↓
Osteogenesis	↑		↑#			
